# *Paulinella acadia* sp. nov., a New Photosynthetic Species Isolated From a Brackish Beach in British Columbia (Canada)

**DOI:** 10.1111/jeu.70040

**Published:** 2025-08-18

**Authors:** Yash Pardasani, Maia V. Palka, Brian S. Leander, Fabien Burki

**Affiliations:** ^1^ Department of Organismal Biology (Systematic Biology), Uppsala University Uppsala Sweden; ^2^ Department of Botany University of British Columbia Vancouver Canada

## Abstract

Plastids in almost all photosynthetic lineages originated from a primary endosymbiosis between cyanobacteria and heterotrophic eukaryotes in an ancestor of Archaeplastida. Strikingly, this event was repeated about a billion years later in an ancestor of photosynthetic *Paulinella*. Due to the recent and independent occurrence of this second primary endosymbiosis, *Paulinella* amoebae serve as a remarkable model group for studying the origin of plastids. To date, three species of photosynthetic *Paulinella* have been described mainly from freshwater and marine environments. Here, we describe a fourth photosynthetic *Paulinella* species from a brackish beach near Vancouver (British Columbia, Canada) using morphological and molecular data that we named *Paulinella acadia* sp. nov. Although *P. acadia* sp. nov. appears similar to *P. chromatophora* under light microscopy, scanning electron microscopy and molecular phylogenetic analyses demonstrate its close relationship to *P. longichromatophora.* The discovery of *P. acadia* sp. nov. expands the diversity and ecological range within this group. Notably, it is the second photosynthetic *Paulinella* species found on a beach to be described, alongside its sister *P. longichromatophora*.

## 1 Introduction

The ability to photosynthesize in eukaryotes has evolved through multiple endosymbiotic events. The point of entry of photosynthesis into the eukaryotic realm was a primary endosymbiosis between a heterotrophic eukaryotic host and a cyanobacterium in an ancestor of the supergroup Archaeplastida. The vast majority of photosynthetic eukaryotes descended from this founding event, either directly in green algae (and land plants), red algae, and glaucophytes, or subsequently via secondary or higher order endosymbioses between eukaryotic cells giving rise to diverse algae, such as ochrophytes, haptophytes, cryptophytes, dinoflagellates, or euglenophytes (Cavalier‐Smith 1998; Palmer 2000; Adl et al. 2005; Burki 2017). The primary endosymbiosis in archaeplastids is an ancient event that likely took place at least 1.5 billion years ago (Sánchez‐Baracaldo et al. 2017; Strassert et al. 2021), making it difficult to reconstruct in detail the steps that transformed free‐living cyanobacteria into an organelle (i.e., organellogenesis). However, another, much more recent primary plastid endosymbiosis that occurred around 60 to 200 million years ago exists in a different supergroup of eukaryotes: the rhizarian *Paulinella* amoebae (Marin et al. 2005; Nowack et al. 2008; Nowack 2014; Macorano and Nowack 2021). The relatively more recent origin of this second primary acquisition of plastids makes *Paulinella* a key model for studying the early stages of primary endosymbiosis (Nowack et al. 2008; Nowack 2014; Macorano and Nowack 2021).

Members of *Paulinella* are pelagic and benthic filose testate amoebae that are found in freshwater, brackish, and marine environments, with a widespread distribution (Yoon et al. 2009; Kim and Park 2016; Lhee et al. 2017; Macorano and Nowack 2021). They exhibit a characteristic oval shape, with a test consisting of silicate scales aligned in columns with a terminal opening (Nicholls 2009). Taxonomically important traits typically include the number of columns of scales (between 3 to 5), the number of scales per column (7–14), the number of oral scales, and the test dimensions (Nicholls 2009; Kim and Park 2016; Lhee et al. 2017). Three photosynthetic species have been described, ranging in length between 11 to 35 μm, and possessing two typical large photosynthetic organelles called chromatophores, which can be either sausage‐shaped or U‐shaped (Kim and Park 2016; Lhee et al. 2017). Although the level of cellular integration of the chromatophores has not always been clear, it is now well‐established that they represent *bona fide* vertically transmitted organelles derived from a cyanobacterium of the *Prochlorococcus*–*Synechococcus* group, distantly related to the cyanobacterial ancestor of plastids in Archaeplastida (Marin et al. 2005; Nowack et al. 2008; Nowack and Grossman 2012; Sørensen et al. 2024). These photosynthetic species are: *P. chromatophora* and *P. micropora*, both cultured and found in freshwater or brackish habitats with salinities up to 10 PSU or 10 g/L (Pankow 1982; Lhee et al. 2017), and *P. longichromatophora*, a marine species not currently available in culture (Kim and Park 2016). Overall, these photosynthetic species have been studied in some detail with genomic, transcriptomic, and even proteomic data (Nowack et al. 2008, 2016; Yoon et al. 2009; Singer et al. 2017; Lhee et al. 2019; Oberleitner et al. 2022). In addition, nine likely heterotrophic species of *Paulinella* have been described, but these remain much more enigmatic (Johnson et al. 1988; Vørs 1993; Hannah et al. 1996; Nicholls 2009). There is no public culture of heterotrophic *Paulinella*, and only one species has been studied with molecular data (Bhattacharya et al. 2012). Varying widely in size from 4.5 μm for *P. ovalis* to 47 μm for *P. gigantica* (Johnson et al. 1988; Nicholls 2009), some of these heterotrophic lineages have been shown to be predators of cyanobacteria and other bacteria (Johnson et al. 1988; Bhattacharya et al. 2012).

Here, we report the morphological and molecular characterization of chromatophore‐bearing *Paulinella* cells isolated from a brackish beach near Vancouver, British Columbia (Canada). Under light microscopy, these cells are morphologically similar to *P. chromatophora* but show chromatophores that are more compact and U‐shaped. Surprisingly, the small and large subunits of the ribosomal DNA genes (18S and 28S rDNA) strongly support a close relationship to *P*. *longichromatophora*, prompting us to propose a new photosynthetic species of *Paulinella* that we named *P. acadia* sp. nov. This discovery brings the number of described photosynthetic species to four and represents the second species described from coastal sediments, alongside *P. longichromatophora*.

## 2 Materials and Methods

### 2.1 Sampling and Cell Isolation

Sediment samples were collected from Spanish Banks, British Columbia, Canada (49.2765° N, 123.2133° W) during low tide in June and July 2024. The samples were transported to the lab, where the cells of interest were concentrated into 60 mm Petri Dishes using the sea ice extraction technique described in (Palka et al. 2025) (adapted from (Uhlig 1964)). Extracted samples were scanned using a Zeiss Axiovert 200 inverted microscope and a Leica DM IL inverted microscope for *Paulinella* cells, and cells of interest were isolated using a hand‐drawn micropipette. *P. acadia* sp. nov. cells were placed on a glass coverslip for differential interference contrast (DIC) microscopy using a Zeiss Axiocam 503 color camera prior to preparation for electron microscopy, confocal laser scanning microscopy, and PCR.

### 2.2 Light and Fluorescence Microscopy

*P. acadia* cells isolated for confocal laser scanning microscopy (CLSM) were prepared on a glass slide in 0.2 μm filtered seawater using a 22 mm 1.5 coverslip. Confocal laser scanning microscopy of prepared slides containing live cells was performed using an Olympus FV1000 confocal laser scanning microscope equipped with a 60× water immersion magnification lens (N.A. 1.20) at the UBC Bioimaging Facility (University of British Columbia). Autofluorescence of the *P. acadia* chromatophores was visualized using an excitation wavelength of 559 nm at 15% transmissivity. Light micrographs were taken with a Leica DM IL inverted microscope at 630× magnification and a Sony alpha7R camera. Micrographs were processed using FIJI (Schindelin et al. 2012) and Affinity Photo (version 1.10.6).

### 2.3 Scanning Electron Microscopy

*P. acadia* cells isolated for scanning electron microscopy were placed directly onto a 0.2 μm Isopore membrane filter (Millipore Sigma) in filtered seawater that was contained within a 13 mm Swinnex filter holder (Millipore Sigma). Cells were vapor‐fixed using 4% osmium tetroxide (Electron Microscopy Sciences) for 10 min at room temperature prior to being washed once in 30% EtOH. Dehydrating and critical point drying of membrane filters containing fixed cells was performed using the methods and equipment described in Palka et al. (2025). Dried membranes containing fixed cells were mounted onto aluminum stubs prior to sputter coating with 2 nm of gold/palladium using a Leica EM ACE200 Low Vacuum Coater. *P. acadia* cells were visualized using a Zeiss XB350 Crossbeam SEM in the Bioimaging Facility (University of British Columbia).

### 2.4 DNA Amplification and PCR

Single isolated cells were picked using pulled glass microcapillaries and washed in filtered seawater. The cells were passed through droplets of minimal seawater to reduce contamination and were added to 0.2 mL PCR tubes with 3 μL of storage buffer provided in the REPLI‐g Advanced DNA Single Cell Kit (Qiagen). The cells were subjected to whole genome amplification using default conditions. The product of MDA reactions was diluted 100× and used as a template in PCR amplification of 18‐28S rDNA genes using *Paulinella* specific forward and reverse primers 5′‐CTATGCGAGGATCCACTGGA‐3′ and 5′‐ACCCTATCTCCTGCTAAACAG‐3′, respectively. These primers were designed using the oligoN‐design pipeline (https://github.com/MiguelMSandin/oligoN‐design/blob/main/README.md), using all available *Paulinella* sequences from the literature. PCR was performed with Takara LA Taq polymerase (Takara) and 2 μL of diluted MDA product as a template. PCR cycling conditions included an initial denaturation step at 94°C for 5 min, 30 cycles of denaturation at 98°C for 10 s, primer annealing at 59°C for 30 s and elongation at 68°C for 4 min, finishing with a final elongation step at 68°C for 5 min. The PCR products were cloned into *E. coli* competent cells using the Topo TA cloning kit (Invitrogen by Thermofisher Scientific). The colonies were picked and grown overnight in 5 mL of TB media with Kanamycin. Plasmids were extracted from 1 mL pellet cells using the miniprep kit (Qiagen) and sequenced on an Oxford Nanopore platform with the Rapid barcoding kit 96, kit 14 chemistry (Oxford Nanopore Technologies, UK). Sequencing was performed by Macrogen (Netherlands).

In addition to MDA, five cells were also picked and placed into lysis buffer of the Smart‐seq2 protocol as described in (Picelli et al. 2014). Following cDNA generation using the Smart‐seq2 protocol described in Picelli et al. 2014, we used 1 μL as template to amplify the 18S rDNA using the same forward primer as above and the *Paulinella* specific reverse primer 5′‐ATTACCCAGGCCTTTCGAGC‐3′, designed using Geneious Prime version 2025.0.3. PCR conditions were the same as above but with an annealing time of 1 min 30 s. The sequences were cloned as described above, and colony PCR products from the picked colonies were sent for Sanger sequencing (Macrogen, Netherlands). cDNA was initially generated for transcriptome work, which is ongoing. Despite targeting poly‐A mRNA, rRNA is abundant and amplifiable with the Smart‐seq2 protocol, this allowed us to successfully amplify 18S from the Smart seq data.

A total of ten cloned sequences (seven from PCR on MDA product and three from PCR on Smart‐seq2 product) were obtained for 18S rDNA, and seven cloned sequences were obtained for 28S rDNA. The sequences obtained were inspected and annotated in Geneious Prime version 2025.0.3.

### 2.5 Phylogenetic Analysis

The new sequences were used for phylogenetic analysis along with all available 18S and/or 28S rDNA sequences for described *Paulinella* species, as well as environmental diversity from GenBank. *Euglypha* was used as an outgroup. The sequences were aligned using MAFFT version 7.490 and gently trimmed with a gap threshold of 0.01 using trimAl version 1.2rev59. IQTREE version 2.2.2.6 was used to infer Maximum Likelihood trees using the best fitting model of nucleotide substitutions TN + F + R2 (for 18S rDNA phylogeny) and TIM2 + F + G4 (for 28S rDNA phylogeny), chosen according to the BIC criterion. The branch support was assessed with standard nonparametric bootstrap using 1000 bootstrap replicates. Pairwise identity comparisons between the new sequences and previously described *Paulinella* species were performed on aligned overlapping regions using Geneious Prime version 2025.0.3.

## 3 Results

*Paulinella acadia* sp. nov.

### 3.1 Occurrence

*Paulinella acadia* sp. nov was isolated from brackish beach sediments collected on Spanish Banks near Vancouver (British Columbia, Canada) during June and July, 2024. The cells were rare and only found occasionally at the sampling site. Single cells were picked and added to *f*/10 media, but attempts to establish a culture have so far been unsuccessful. Salinity at the collection site varies seasonally, ranging from approximately 20 PSU in spring to nearly 30 PSU by late summer, suggesting that *P. acadia* sp. nov. may tolerate a relatively broad salinity range.

### 3.2 Morphology

**FIGURE 1:**
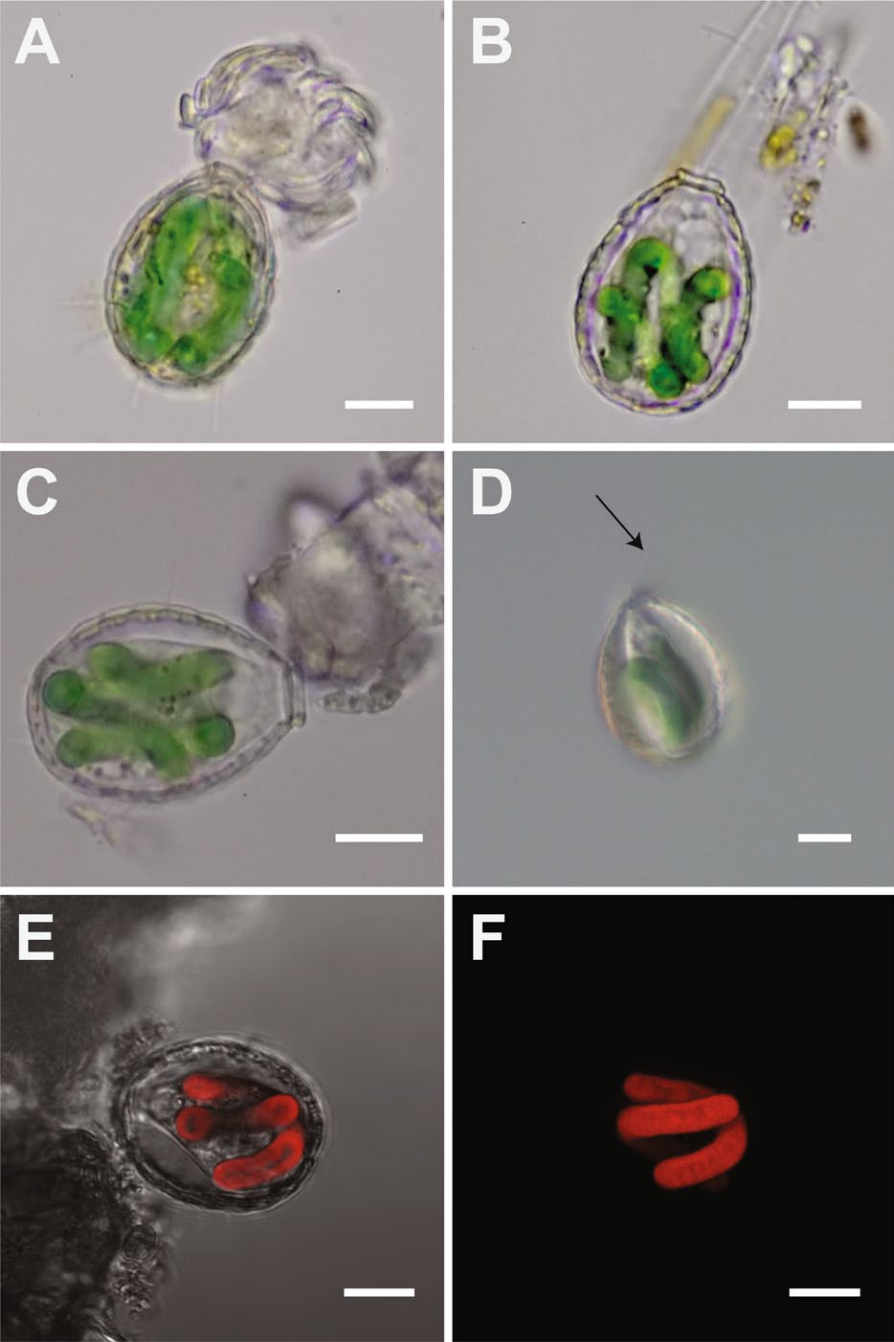
Light and confocal images of *Paulinella acadia* sp. nov. (A–C) Light micrograph images showing two chromatophores, (D) Light micrograph images with DIC, (E, F) Confocal images showing plastid autofluorescence under 559 nm excitation. Black arrow shows the pseudopodia. All scales bar are 10 μm.

**FIGURE 2:**
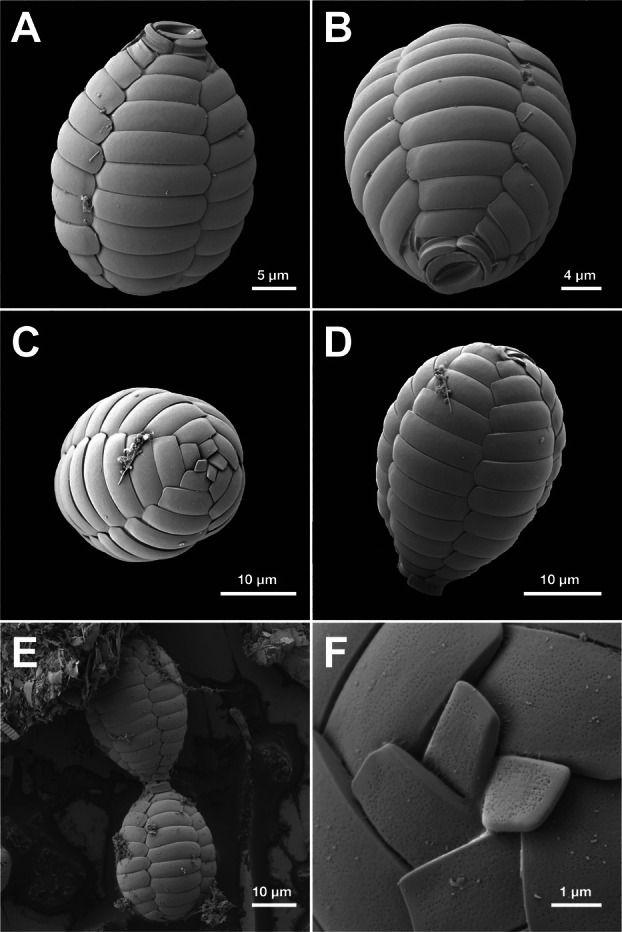
Scanning electron micrographs (SEMs) of *Paulinella acadia* sp. nov. (A, B) Lateral views. (C) Posterior view. (D) Anterior end of test attached to the substrate. (E) Two cells attached by the anterior opening. (F) High magnification SEM showing the scales on the posterior end of the test.

The cells displayed the characteristic oval‐shaped test of *Paulinella* with an opening at the anterior end of the cell from which filose pseudopodia extended (Figure 1A,D). A circular vacuole was observed on the anterior side of the cell. *P. acadia* had two U‐shaped chromatophores (Figure 1E,F); however, in some cells, coiled chromatophores were also observed (Figure 1B,C). In healthy cells, the chromatophores emitted bright red autofluorescence under 559 nm excitation wavelength. Based on one cell, the chromatophores were 3.7 μm in diameter and 31.4 μm long (Figure 1E,F). The anterior opening was surrounded by five oral scales: two oral scales arranged elliptically around the opening with three shorter oral scales surrounding them. The test ranged from 28.8 to 31.9 μm long (*n* = 4) and 21.5–23.9 μm wide (*n* = 7) and was composed of rectangular scales with rounded corners. The corners were smoother on the scales toward the middle of the cell but sharper and more rectangular closer to the anterior end (Figure 2). Scales in the middle of the cell were larger than the scales in the anterior and posterior regions. The body of the test consisted of non‐overlapping scales arranged in five columns with eleven rows of scales per column, and each scale had numerous fine pores present on the external surface throughout, similar to *P. micropora* (Lhee et al. 2017).

### 3.3 Phylogenetic Analysis

**FIGURE 3:**
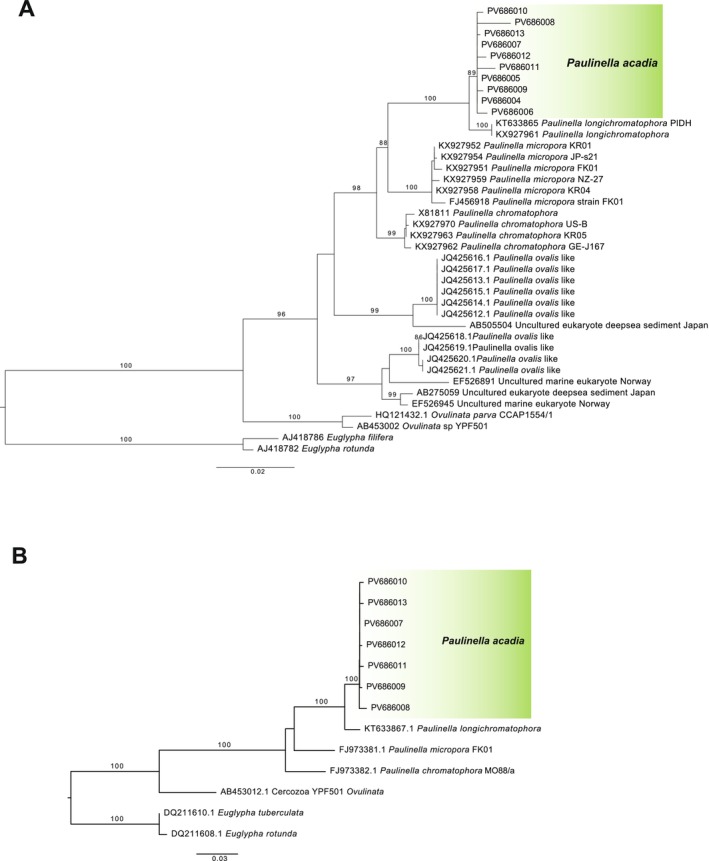
Maximum Likelihood tree inferred from (A) 18S rDNA sequences and (B) 28S rDNA sequences, showing the phylogenetic position of *Paulinella acadia* sp. nov. *Euglypha* sequences were used to root the tree. The clade highlighted in green shows the position of the new *Paulinella acadia* sp. nov. species. Only bootstrap values > 85% are shown on the tree.

A total of ten 18S rDNA sequences (length ranging from 800 bp to 1267 bp) and seven 28S rDNA sequences (1756 bp to 1762 bp) were obtained from individually isolated cells (Table S1). These sequences were combined with available data retrieved from GenBank and subjected to Maximum Likelihood phylogenetic reconstructions. Both trees recovered the new *P. acadia* clone sequences clustering together (89% and 100% bootstrap support based on the 18S rDNA and 28S rDNA trees, respectively) and fully supported as sister to the marine species *P. longichromatophora* (Figure 3). Pairwise identity between *P. acadia* and *P. longichromatophora* ranged from 98.3%–99.2% for 18S rDNA and 96.7%–97.2% for 28S rDNA. The other two photosynthetic species branched as expected from previous phylogenetic analyses, that is, *P. micropora* was sister to the new grouping of *P. acadia* and *P. longichromatophora*, and *P. chromatophora* diverged first (Kim and Park 2016; Lhee et al. 2017) (Figure 3). In the 18S rDNA tree, sequences obtained from single‐amplified genomes of cells superficially resembling *P. ovalis* and a few environmental sequences completed the diversity and branched outside of the photosynthetic clade (Figure 3A).

## 4 Discussion

In contrast to the primary endosymbiosis that occurred in an ancestor of Archaeplastida, which led to the evolution of an estimated 450,000–500,000 living species in this supergroup alone (Bowles et al. 2023), only three photosynthetic species are known to have descended from the primary endosymbiosis in *Paulinella* (Marin et al. 2005; Kim and Park 2016; Lhee et al. 2017). There is no reason to think that *Paulinella* will necessarily follow the successful evolutionary trajectory of Archaeplastida, and the rarity of photosynthetic *Paulinella* species can be expected based on the much shorter timescale that has spanned since this more recent primary endosymbiosis. However, it is possible that other photosynthetic species have remained hidden as a result of the general scarcity of these cells in the environment. Here, we show that more photosynthetic *Paulinella* species exist by describing a novel species.

From routine observations of brackish beach sediment samples, we observed cells that were superficially very similar to *P. chromatophora*, but inhabit a different environment as *P. chromatophora* is known from freshwater ponds and lakes, typically found in shady sediments. Table 1 provides a detailed morphological comparison of the known photosynthetic *Paulinella* species and strains. When observed with light microscopy, *Paulinella acadia* sp. nov. cells show a general resemblance to *P. chromatophora*, particularly the overall oval shape and size dimensions, which helps explain why they have remained hidden in plain sight as a distinct species on a relatively well‐studied beach for protist diversity (Noble et al. 2007; Chantangsi et al. 2008, 2010; Sparmann et al. 2008). However, ultrastructural observations reveal details that clearly distinguish this novel isolate from *P. chromatophora*. They possess five oral scales instead of three, which is the same number as in *P. longichromatophora* and *P. micropora*, and eleven scales per column, again similar to these species. Chromatophore morphology also contributes to distinguishing these species: *P. acadia* sp. nov. and *P. longichromatophora* both have U‐shaped chromatophores, whereas *P. chromatophora* and *P. micropora* exhibit a less pronounced bend and more sausage‐like chromatophores (Marin et al. 2005; Yoon et al. 2009; Kim and Park 2016; Lhee et al. 2017). Our phylogenetic reconstruction is consistent with these morphological observations, placing together *P. micropora* and the new clade consisting of *P. longichromatophora* and *P. acadia* sp. nov. (Figure 3). Further supporting the similar scale number and organization, both *P. acadia* sp. nov. and *P. longichromatophora* are found in similar coastal environments consisting of fine sandy beaches, although they appear to occupy different salinity regimes. *P. acadia* sp. nov. was found in a brackish beach with salinity ranging from 20 PSU to 27 PSU, whereas *P. longichromatophora* has been reported from a marine beach with higher salinity between 29 and 30.6 PSU (Kim and Park 2016). This ecological distinction may reflect differences in salinity tolerance or habitat preference between these two species. Despite their close phylogenetic relationship, both species also differ in the shape of the test. *P. longichromatophora* is more elongate (27–35 μm long and 14–19 μm wide), whereas *P. acadia* sp. nov. is shorter and broader (28.8 to 31.9 μm long and 21.5–23.9 μm wide), resulting in a noticeably different length‐to‐width ratio (Kim and Park 2016). Another distinguishing feature between these two sister species is the scale surface structure; *P. longichromatophora* has a smooth external surface (Kim and Park 2016) whereas *P. acadia* sp. nov. has numerous fine pores present on the external surface (Table 1).

**TABLE 1:** Comparison of morphological characters among the photosynthetic *Paulinella* species.

Feature	*Paulinella acadia* sp. nov.	*Paulinella longichromatophora*	*Paulinella micropora* KR01	*Paulinella* FK01	*Paulinella micropora* NZ‐27	*Paulinella chromatophora* CCAC 0185	*Paulinella chromatophora* NZ‐13.11
Habitat	Marine sediment (20.0 to 27.0) psu)	Marine sediment (29.0 to 30.6 psu)	Fresh water	Fresh water	Fresh water	Fresh water	Fresh water
Chromatophore morphology	U shaped	Two U shape; sometimes one U shape and 1 rod shape	Sausage shaped	Sausage shaped	Sausage shaped	Sausage shaped	Sausage shaped
Length (μm)	28.8–31.9	27–35	11.5–13.8	15–17	15.8	23–27	25.8–29.4
Width (μm)	21.5–23.9	14–19	9.1–10.7	10–11	12.7	16–20	19.4–19.8
Average Length/Width Ratio	30.35/22.715	31/16.5	12.65/9.9	16/10.5	15.8/12.7	25/18	27.6/19.6
Length/Width	1.336	1.878	1.277	1.523	1.244	1.388	1.408
Oral scales	5	5	5	5	5	3	3
No of columns	5	5	5	5	5	5	5
No of scales per column	11	10–12	8–9	10–11	10	12–14	12–14
Scale features^a^	Fine pores ext.	Smooth ext.; sieve‐plate pores int.	Fine pores ext.; basal pustules; sieve‐plate pores int.	Fine pores ext.; basal pustules; sieve‐plate pores int.	Fine pores ext.; basal pustules; sieve‐plate pores int.	Postules ext.; sieve‐plate pores int.	Fine pores ext.; postules on bottom of ext.

^a^ ext., external; int., internal.

The unique combination of the molecular phylogenetic placement, morphology, and environmental niche of this new BC isolate suggests that it represents a novel species of *Paulinella*, probably recently diverged from *P. longichromatophora*, resulting in low genetic divergence, making it the fourth photosynthetic species in the genus. We named it *Paulinella acadia* sp. nov., after Acadia beach, the locality where the cells were discovered.

The more recent primary endosymbiotic event that occurred in photosynthetic *Paulinella* provides a unique opportunity to further elucidate the fundamental processes that result in the transition from endosymbiont to organelle. The discovery of additional photosynthetic diversity within the genus *Paulinella* is thus relevant to better understand the genomic processes that follow primary endosymbiosis. The addition of this fourth photosynthetic species represents the first described brackish coastal species. Future work will aim to establish a culture of the new species and, in priority, obtain the full chromatophore genome sequence.

### 4.1 *Paulinella acadia* sp. nov. Pardasani, Palka, Leander and Burki

Diagnosis: Oval‐shaped test, 28.8–31.9 μm long, 21.5–23.9 μm wide, composed of siliceous rectangular scales with rounded corners arranged in 5 columns with 11 scales per column, with an anterior opening. The cells possess two U‐shaped chromatophores, 3.7 μm in diameter and 31.4 μm in length.

Holotype: Collection of specimens on SEM stub is deposited at the Beaty Biodiversity Museum in Vancouver, British Columbia, Canada under the accession number MI‐PR227.

Type habitat: Marine sediments of Spanish Banks, British Columbia, Canada.

Type locality: Spanish Banks, British Columbia, Canada (49.2765° N, 123.2133° W).

Etymology: The species is named *acadia* after Acadia Beach, a close locality to where the cells were discovered.

Gene sequences: Sequences of nuclear 18S and 28S rDNA regions used for this study have been deposited in GenBank under accession numbers PV686004–PV686013.

Zoobank registration: Described under the zoological code; Zoobank registration urn:lsid:zoobank.org:act:BEF292EF‐A9E7‐44DD‐9854‐675F6002B35C.

This publication (work) has been registered with zoobank as: urn:lsid:zoobank.org:pub:2C68C4BD‐01B9‐4A76‐9BEC‐4B4CEBE6DF2D.

## Supporting information


**Table S1:** Genbank accession numbers of all sequences obtained during this study, with corresponding amplified rDNA region and primers used for PCR.

## Data Availability

The data that support the findings of this study are openly available in NCBI GenBank at https://www.ncbi.nlm.nih.gov/genbank/, reference number PV686004‐PV686013.
