# Supplementary material for: Paulinella acadia sp. nov., a New Photosynthetic Species Isolated From a Brackish Beach in British Columbia (Canada)
Source: J Eukaryot Microbiol. 2025 Aug 18;72(5):e70040. doi: 10.1111/jeu.70040 (PMC12358764; doi:10.1111/jeu.70040)
Supplement: Supplementary file 1 — Table S1: Genbank accession numbers of all sequences obtained during this study, with corresponding amplified rDNA region and primers used for PCR. [file JEU-72-e70040-s001.docx]

| #Accession | Region | Forward primer used | Reverse primer used |
| --- | --- | --- | --- |
| PV686004 | 18S | 5’-CTATGCGAGGATCCACTGGA-3’ | 5’-ATTACCCAGGCCTTTCGAGC-3’ |
| PV686005 | 18S | 5’-CTATGCGAGGATCCACTGGA-3’ | 5’-ATTACCCAGGCCTTTCGAGC-3’ |
| PV686006 | 18S | 5’-CTATGCGAGGATCCACTGGA-3’ | 5’-ATTACCCAGGCCTTTCGAGC-3’ |
| PV686007 | 18S,28S and ITS between them | 5’-CTATGCGAGGATCCACTGGA-3’ | 5’-ACCCTATCTCCTGCTAAACAG-3’ |
| PV686008 | 18S,28S and ITS between them | 5’-CTATGCGAGGATCCACTGGA-3’ | 5’-ACCCTATCTCCTGCTAAACAG-3’ |
| PV686009 | 18S,28S and ITS between them | 5’-CTATGCGAGGATCCACTGGA-3’ | 5’-ACCCTATCTCCTGCTAAACAG-3’ |
| PV686010 | 18S,28S and ITS between them | 5’-CTATGCGAGGATCCACTGGA-3’ | 5’-ACCCTATCTCCTGCTAAACAG-3’ |
| PV686011 | 18S,28S and ITS between them | 5’-CTATGCGAGGATCCACTGGA-3’ | 5’-ACCCTATCTCCTGCTAAACAG-3’ |
| PV686012 | 18S,28S and ITS between them | 5’-CTATGCGAGGATCCACTGGA-3’ | 5’-ACCCTATCTCCTGCTAAACAG-3’ |
| PV686013 | 18S,28S and ITS between them | 5’-CTATGCGAGGATCCACTGGA-3’ | 5’-ACCCTATCTCCTGCTAAACAG-3’ |
